# Sinister right-handedness provides Canadian-born Major League Baseball players with an offensive advantage: A further test of the hockey influence on batting hypothesis

**DOI:** 10.1371/journal.pone.0221501

**Published:** 2019-08-29

**Authors:** Denver M. Brown, Zoe A. Poucher, Matt Myers, Jeffrey D. Graham, John Cairney

**Affiliations:** 1 Faculty of Kinesiology & Physical Education, University of Toronto, Toronto, Ontario, Canada; 2 Institute of Biomaterials and Biomedical Engineering, University of Toronto, Toronto, Ontario, Canada; 3 Department of Family Medicine, McMaster University, Hamilton, Ontario, Canada; Fundació Mona, SPAIN

## Abstract

Recent research has shown Major League Baseball (MLB) players that bat left-handed and throw right-handed, otherwise known as sinister right-handers, are more likely to have a career batting average (BA) of .299 or higher compared to players with other combinations of batting and throwing handedness. Moreover, possibly owing to early exposure to hockey, Canadian-born MLB players have an increased propensity to be sinister right-handers, however, it has yet to be determined whether this provides a relative offensive performance advantage compared to players born in other countries. Using the largest archival dataset of MLB statistics available, the present study examined the independent influence of batting (i.e., left, right, switch) and throwing (i.e., left, right) handedness combinations and country/region of origin (i.e., Canada, USA, Latin America, Asia, Other) on several indices of offensive performance including BA, slugging percentage (SLG), on-base plus slugging (OPS), on-base plus slugging plus (OPS+), home runs (HR), runs batted in (RBI), strikeouts (SO) and wins above replacement (WAR). Mediation models were also computed to examine whether birthplace influences offensive performance through handedness. Examination of all recorded MLB batters revealed that batting left, regardless of throwing handedness, confers an offensive performance advantage. Since the inception of the MLB, the relative proportion of Canadian-born sinister right-handers is at least two times greater than players from other regions, although being Canadian-born does not provide a direct offensive advantage. Rather, results showed evidence of a significant indirect effect in that being Canadian-born increases the odds of being a sinister right-hander and in turn leads to greater performance across each offensive performance statistic. Collectively, findings provide further support for the hockey influence on batting hypothesis and suggest this effect extends to offensive performance.

## Introduction

Left-handed batting in baseball gives players an advantage over right-handed batters, evidenced by the fact that left-handed batters are more likely to win batting titles than right-handed batters [1]. Several hypotheses have been advanced to explain this finding. For instance, left-handed players are a step closer to first base and the momentum of their swing is orientated in the direction of the run, making it more likely they will beat out close throws [2]. Additional explanations include the novelty or limited experience pitchers have facing left-handers, the increased chance of facing right-handed pitchers (so called “off-handed” matchups), and the possibility to capitalize on shifts in field position. From a neurocognitive perspective, hemispheric lateralization has also been advanced as an explanation [3]. Generally, left-hand dominant individuals show less lateralization than right-hand dominant individuals meaning there is less differentiation in function by hemisphere. This greater flexibility may manifest in advanced skill performance. McLean and Ciurczak [3] first applied this to baseball and were able to demonstrate that dominant left-handed players (players who throw and bat left) were more likely to play in the major leagues and among professional players, were three-times more likely to have career batting averages (BA) over .299. Grondin et al. [2] extended this line of inquiry by examining differences between handedness preference orientations for BA as well as advanced batting performance metrics that reflect power hitting (i.e., slugging percentage [SLG]; home runs [HR]) and plate discipline (i.e., strikeouts [SO], walks). Using a dataset from the inception of Major League Baseball in 1871 to the end of the 1993 season, their results showed dominant left-handed players have higher BAs and more walks than dominant right-handed players, while also batting for a higher SLG and more HRs per at bat than players with all other combinations of throwing and batting handedness preference orientations. However, batting for more power did come at the expense of recording a SO more often in comparison to left-handed batters who throw right-handed. Grondin et al. [2] provided a potential explanation for their findings, in that dominant left-handed batters execute a forehand stroke (as in tennis) which provides biomechanical (i.e., spring effect) and kinematic (i.e., increased peak swing velocity) advantages that facilitate more power as opposed to a less powerful, more reliable backhand stroke that is executed by left-handed batters who throw right-handed.

Recently, Mann, Loffing, and Allen [4] updated and re-analyzed the data originally used by McLean and Ciurczak [3] and found that the biggest advantage in batting was not for dominant left-handed players, but for left-handed batters who throw right-handed, also known as sinister right-handed players. In their analysis, sinister right-handers were more than seven-times more likely to play in the majors relative to the proportion of other combinations of throwing and batting handedness preferences in a representative sample of 538 high school and grammar school students, and more than three and half-times more likely than dominant left-handed batters to have a career BA over .299. The authors suggest that this finding casts doubt upon McLean and Ciurczaks’s [3] proposition that the offensive advantage of dominant left-handed players is attributable to hemispheric laterization. Instead, Mann et al. [4] postulate a biomechanical advantage for sinister right-handers whereby the dominant hand ends up being positioned further from the striking end of the bat, which gives greater leverage and more power in the swing (while also noting it may result in less bat control)[4]. However, the only offensive performance statistic Mann et al. [4] assessed was BA, which would be considered more indicative of bat control than power. Inferring that sinister right-handers have a power advantage directly contradicts findings of Grondin et al. [2], which showed dominant left-handed players hit more HR per at bat and have a higher SLG in comparison to sinister right-handers. Given the difference in findings observed by Mann et al. [4] over 35 years after the original study of McLean and Ciurczak [3], it is also possible that the earlier findings of Grondin et al. [2] will not be supported over twenty-five years later. Findings will lend insights as to whether certain handedness preference orientations provide specific offensive performance advantages for MLB players.

While there is little doubt that there is an advantage to batting left-handed in terms of BA, BA itself is not a particularly sensitive or even accurate measure of offensive performance. Previous research examining advanced offensive performance metrics such as HR and SLG have improved our understanding of potential handedness-based offensive performance advantages [2], however additional advanced offensive statistics will offer further insight regarding this knowledge gap. Notwithstanding the contradicting theories for possible biomechanical advantages of left-handed batters who throw right-[4] and left-handed[2], many of the explanations offered to explain the relative advantage involve more than just the mechanics of the swing. Walks, for example, are just as valuable as hits for getting on base. Situational hitting, that is, adjusting approach at the plate depending on outs and whether or not there are players already on base is also important to evaluate offensive performance. For example, a player that is on base is more likely to advance to the next base on a ball that is hit in play than attempting to steal a base on a SO. Many of the aforementioned explanations, such as capitalizing on right-left matchups in pitching, and adjusting to shifts in field position, suggest reading and reacting to the field are also important. Sinister right-handed batters may be better able to read off-speed and breaking pitches because their dominant eye is closer to the pitcher[5]. This of course assumes they are also right-eye dominant.

Although previous studies have relied on outcomes like lifetime BA [2,4] and SLG [2], these are very limited measures of performance. In the former case, BA is simply the number of times a player records a hit divided by the number of times at the plate. BA does not reflect a players’ ability to draw walks nor does it take into account the type of hit a player makes (e.g. single versus double, triple or homerun). BA is also affected (as are measures of hitting in general) by environmental conditions such as the dimensions of the field (distance from home plate to the outfield wall–right, left and centre field), as well as the altitude of the park. To overcome the limitations of BA, SLG takes into account the productivity of the hitter by giving more weight to extra base hits (e.g., doubles). The equation is: SLG = (((1 * 1B) + (2 * 2B) + (3 * 3B) + (4 * HR))/AB). Where B represents the base, AB is at bats and HR is home runs. As can been seen in the formula, a simple multiplicative factor is included to give relatively more weight to hits that advance the runner further around the bases. However, a major shortcoming of SLG is that this advanced statistic does not account for ‘walks’.

On-base plus slugging (OPS), as the title suggests, adds SLG to on-base percentage (OBP); because OBP includes both hits and walks, OPS provides a more complete accounting of offensive performance. A further adaption, denoted as OPS+, takes into account the influence of different parks on performance. The equation is: OPS+ = 100 * ((OBP / *lgOBP) + (SLG / *lgSLG)– 1). Where *lgOBP and *lgSLG are park-adjusted league OBP and SLG (excluding pitchers hitting performance), respectively. By taking into consideration relative advantages attributable to playing in specific parks, OPS+ is considered an improvement over OPS.

Wins above replacement (WAR) is a summary statistic that is intended to reflect a players’ total contribution to the team. Specifically, the value for WAR represents the number of additional wins the team has achieved above expected wins if the player in question was replaced by a minor leaguer or bench player. Positive values (>0) suggest the player in question contributed more to wins than a replacement player. Although there are some variations in how the components of the formula are calculated, the general form of the equation is: WAR = (Batting Runs + Base Running Runs + Fielding Runs + Positional Adjustment + League Adjustment + Replacement Runs) / (Runs Per Win).

Finally, runs batted-in (RBI) is also an extremely useful offensive statistic, particularly in the current era where hitting for power is dominant. Despite the fact that RBI is widely regarded as more valuable to evaluate player performance than statistics such as BA, research to date on left-handed batting has not examined whether left-handed hitters, including sinister right-handers, outperform other right-handed batters.

Beyond advantages to BA (and possible advantages to other performance statistics), selection of left-handed hitters (both dominant left-handers and sinister right-handers) into the MLB itself is not a random process. Given the advantages of batting left-handed, we might expect for example that left-handed hitters would be selected at a higher rate than right-handed players. Although the proportion or prevalence of left-handed batters in the majors exceeds that which would be expected given the number of left-handed people in the general population [6,7], some countries contribute a higher relative number of left-handed hitters to the pool of players. Cairney and colleagues [5] recently showed that Canadian-born MLB players were far more likely to bat left than players born in the United States, Dominican Republic or South-east Asia (69% compared to 37%, 33% and 30%, respectively). Interestingly, more than 60% of left-handed Canadian-born players active at the time of the study threw right-handed. The explanation for this was linked to ice hockey–a game widely played in Canada. Unlike baseball, hand dominance is much less predictive of bimanual control (i.e., shooting) patterns in hockey [7]. Considered in this context, for those who shoot with their right-hand at the top of the stick, it is a fairly natural transition to place that same hand at the bottom of the baseball bat, resulting in a left-handed swing. According to Cairney et al. [5], coaches and other analysts of the game have speculated that playing hockey first, early in life, produces a higher prevalence of left-handed batters in baseball emanating from this country. Given the advantages to batting left-handed previously noted, Canadian-born left-handed batters are likely to be selected into the MLB at higher rate than players from other countries. At present however, it is not known if Canadian-born baseball players outperform American-born players and those from other countries on the advanced performance statistics identified above.

Given the aforementioned gaps in our knowledge regarding a relative advantage for sinister right-handers, this study used archival data to examine the following research questions. (1) Are there differences in offensive performance across different groups defined by handedness preference with regard to throwing and batting handedness? (2) Do Canadian-born players outperform American-, Latin-American- and Asian-born players as well as players born anywhere else on offensive statistics? (3) Are Canadian born players more likely to bat left-handed and throw right-handed compared to American-, Latin-American-, Asian-born and players born anywhere else? (4) If Canadian-born MLB players have a greater propensity to bat left-handed and throw right-handed, does this provide a relative offensive performance advantage compared to players coming from other places of origin?

## Methods

### Data extraction

The online database baseballreference.com was used to extract country of birth, handedness preference for batting and throwing, and offensive performance (i.e., BA, SLG, OPS, OPS+, HR, RBI, SO, WAR) for each position player (i.e., non-pitchers) to record at least one at bat in an MLB game since the leagues inception to the end of the 2018 season. Players listed as both pitchers and position players were included in the sample. Given that the quantity of at bats a player receives influences the number of HR, RBI and SO they can potentially accrue, we computed a rate of production (e.g., the percentage of at bats in which they recorded a HR) for these statistics using the following formula with HR as an example: (total HR / total at bats) * 100. To ensure the reliability of the data obtained from the baseballreference.com database, 1% of the sample was randomly selected via SPSS to have BA, SLG, OPS and RBI cross-referenced against the mlb.com online database. Results of the reliability analysis revealed 94.5% accuracy of which 1.75% (*n* = 7) of cases had a discrepancy of ≤.01 units, and 2.75% (*n* = 11) of cases on mlb.com were missing data, likely due to the historical nature of the statistics (e.g., 1873–1876 and 1902 seasons). For country of birth, all players were classified based on the location they were born. Players were then grouped based on being born in Canada, USA, Latin-America (i.e., Mexico and Central American, Caribbean and South American countries), Asia (i.e., Japan, South Korea, Taiwan, Hong Kong, Afghanistan, Philippines, China, Saudi Arabia, Vietnam, Singapore) and Other (i.e., anywhere else). Given that all of the data for this analysis is publicly available, and that we do not report any individual names of players or report samples sizes less than 5, research ethics approval on this project was not required.

### Data analysis

Descriptive statistics were computed for all study variables. To evaluate our hypotheses regarding whether there are differences in offensive performance for handedness and birthplace, separate one-way analyses of variance (ANOVAs) were computed with post-hoc (Games-Howell) tests to decompose significant effects. To control for the potential of a high false discovery rate due to the number of post-hoc comparisons (≥ 10) [8], Benjamini-Hochberg adjustment was used [9]. Estimated effect sizes for the overall analyses are reported as partial eta squared (η_p_^2^).

**Fig 1:**
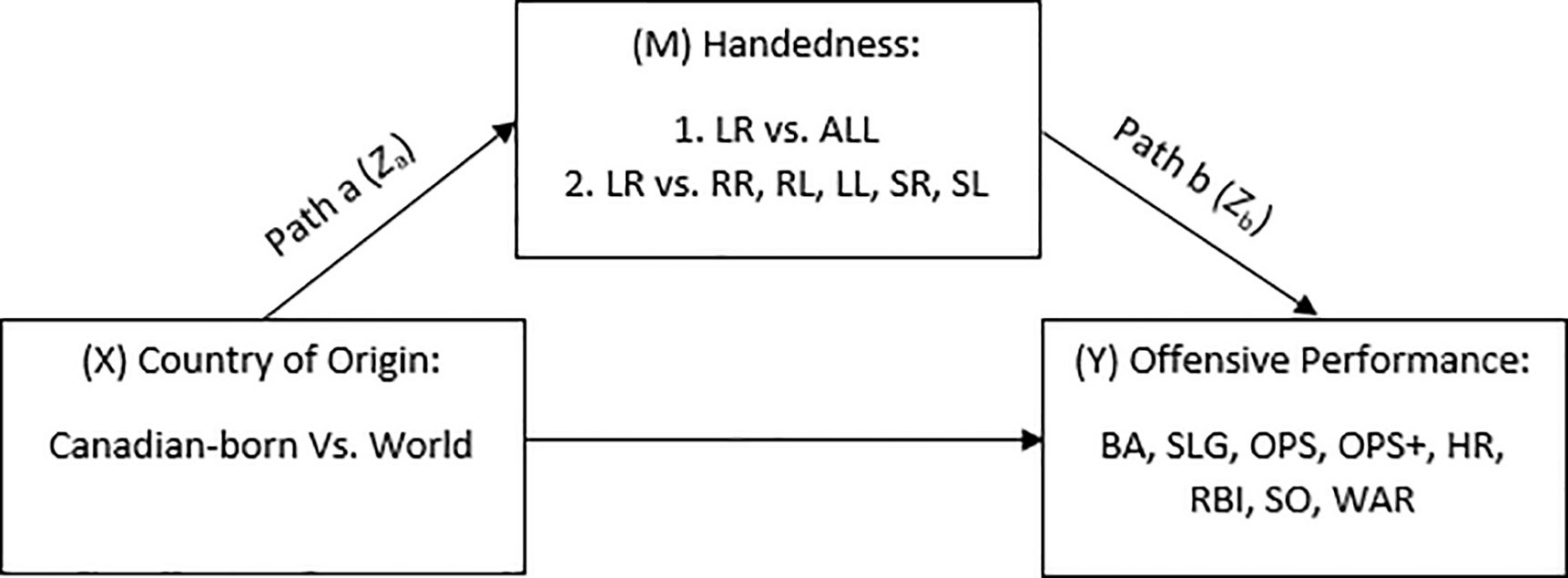
Proposed Mediational Pathway by which Country of Origin Influences Offensive Performance through Handedness in Batting and Throwing. LR = throws left, bats right; LL = throws and bats left; RR = throws and bats right; RL = throws right, bats left; SR = bats left and right, throws right; SL = bats left and right, throws left; BA = batting average; SLG = slugging percentage; OPS = on-base plus slugging; OPS+ = on-base plus slugging plus; HR = home runs; RBI = runs batted in; SO = strikeouts; WAR = wins above replacement.

Given that bivariate analysis techniques (i.e., ANOVA) do not account for potential mediating variables such as the influence of birthplace on offensive performance through handedness, further analyses were conducted. First, a chi-square test was computed to examine whether there are greater proportion of Canadian-born sinister right-handers than players from the USA, Latin America, Asia and anywhere else. The estimated effect size for the chi-square test is reported as Cramer’s V. Next, to test our hypothesis regarding whether an increased likelihood for Canadian-born players to be sinister right-handers provides a relative performance advantage compared to players from other countries, a series of mediation models (see Fig 1) were computed using Iacobucci’s [10] methodological extension for computing mediation using different combinations of continuous and categorical X, M and Y variables. This method allows for comparison of ordinary least squares and logistic regressions using a *Z*-test to control for differences in scale among regression coefficients. In the present study, our X (birthplace) and M (handedness) variables were categorical, whereas our Y (offensive performance indices) variables were continuous. Following methods outlined by Iacobucci [10], the first step involved computing a logistic regression model to examine whether birthplace predicts handedness (path a). The second step involved computing a linear regression model to examine whether handedness predicts offensive performance (path b) when controlling for birthplace. The third step involved computing the standardized elements for path a (*Z*_a_) and path b (*Z*_b_) by dividing each parameter estimate by its respective standard error. The final step involved computing a *z*-test by dividing the product of the standardized elements (*Z*_a_*Z_b_) by their collected standard error (√(*Z*_a_^2^ + *Z*_b_^2^ +1)) and comparing it against a standard normal *z*-score value of |1.96| at α = .05. For step 1, dummy coding was used to assign numerical values to Canada (1) and all countries except Canada (0). For step 2 in the first mediation model, dummy coding was used to assign numerical values to sinister right-handers (1) and all handedness patterns except sinister right-handers (0). For step 2 in the second series of mediation models, dummy coding was used to assign numerical values to sinister right-handers (1) and each of the other handedness patterns (0), respectively. All statistical analyses were performed using IBM SPSS Version 25 with the exception of Iacobucci’s mediation method, which was computed using a custom macro in Microsoft Excel 2016.

## Results

### Handedness

Offensive performance values are displayed by group in Table 1. The first step in our analysis plan involved testing whether handedness influences MLB players’ offensive performance. Results of separate one-way ANOVAs revealed significant between-groups differences for BA, *F* (5, 9769) = 4.67, *p <* .001, η_p_^2^ = .00, SLG, *F* (5, 9769) = 6.85, *p* < .001, η_p_^2^ = .00, OPS, *F* (5, 9769) = 10.18, *p* < .001, η_p_^2^ = .01, OPS+, *F* (5, 9769) = 9.77, *p* < .001, η_p_^2^ = .01, HR, *F* (5, 9769) = 4.85, *p <* .001, η_p_^2^ = .00, RBI, *F* (5, 9736) = 4.06, *p* = .001, η_p_^2^ = .00, SO, *F* (5, 9700) = 4.18, *p* = .001, η_p_^2^ = .00, and WAR, *F* (5, 9769) = 6.86, *p* < .001, η_p_^2^ = .00. Levene’s test indicated unequal variances among the groups for each offensive performance category (all *ps* < .05), therefore, Games-Howell post-hoc tests with Benjamini-Hochberg adjustment were computed and are displayed in Table 1.

**Table 1:** Offensive performance values by handedness orientation.

	Handedness					
Offensive Performance Category	Bats right, throws right (RR) *n* = 5884 *M* (SD)	Bats right, throws left (RL) *n* = 71 *M* (SD)	Bats left, throws left (LL) *n* = 1233 *M* (SD)	Bats left, throws right (LR) *n* = 1736 *M* (SD)	Bats right and left, throws right (SR) *n* = 796 *M* (SD)	Bats right and left, throws left (SL) *n* = 55 *M* (SD)
BA	.226 (.09)_a_	.228 (.07)	.236 (.08)_a_	.234 (.08)_b_	.231 (.08)	.241 (.05)
SLG	.312 (.14)_ab_	.332 (.12)	.338 (.15)_ac_	.332 (.12)_b_	.321 (.12)_c_	.333 (.09)
OPS	.602 (.22)_ab_	.614 (.18)	.638 (.20)_a_	.634 (.19)_b_	.619 (.20)	.651 (.15)
OPS+	66.77 (60.56)_abc_	70.76 (48.75)	76.30 (55.03)_ad_	75.16 (52.19)_b_	69.79 (53.68)_d_	80.78 (37.28)_c_
HR^%^	1.34 (1.84)_a_	1.56 (1.91)	1.54 (1.80)_ac_	1.45 (1.63)_b_	1.22 (1.34)_bc_	1.26 (1.45)
RBI^%^*	9.74 (6.48)_a_	10.26 (5.23)	10.19 (5.84)_c_	9.95 (5.51)_b_	9.01 (4.84)_abc_	9.75 (4.20)
SO^%^^†^	17.76 (13.17)_a_	15.96 (11.08)	17.93 (13.06)	16.64 (12.22)_ab_	18.93 (11.20)_b_	18.08 (9.83)
WAR	4.72 (12.44)_ab_	3.59 (14.66)	6.27 (15.54)_a_	6.44 (14.54)_b_	6.04 (13.47)	6.73 (14.98)

Note: Values in each row that share the same subscript indicate significant between-group differences using Benjamini-Hochberg adjustment.

^%^ = Percentage of at bats ([Category/Total At Bats] x 100).

* = samples sizes for RBI: RR (n = 5859), RL (n = 71), LL (n = 1229), LR (n = 1733), SR (n = 795), SL (n = 55).

^†^ = samples sizes for SO: RR (n = 5837), RL (n = 71), LL (n = 1224), LR (n = 1724), SR (n = 795), SL (n = 55).

### Birthplace

The next step of our analysis involved testing whether country of origin influences MLB players’ offensive performance. Offensive performance values are displayed by group in Table 2. Results of separate one-way ANOVAs revealed significant between-groups differences for BA, *F* (4, 9770) = 3.24, *p =* .012, η_p_^2^ = .00, SLG, *F* (4, 9770) = 8.26, *p* < .001, η_p_^2^ = .00, OPS, *F* (4, 9770) = 3.97, *p* = .003, η_p_^2^ = .00, HR, *F* (4, 9770) = 19.75, *p* < .001, η_p_^2^ = .01, and SO, *F* (4, 9701) = 12.59, *p* < .001, η_p_^2^ = .01, however, there were no differences for OPS+, *F* (4, 9770) = 1.06, *p* = .37, η_p_^2^ = .00, RBI, *F* (4, 9737) = 1.51, *p* = .20, η_p_^2^ = .00, and WAR, *F* (4, 9770) = .07, *p* = .99, η_p_^2^ = .00. Levene’s test indicated unequal variances among the groups for each offensive performance category (all *ps* < .05) except WAR, therefore, Games-Howell post-hoc tests with Benjamini-Hochberg adjustment were computed and are displayed in Table 2.

**Table 2:** Offensive performance values by birthplace.

	Birthplace				
Offensive Performance Category	Canadian-born *n =* 110 *M* (SD)	USA-born *n =* 8469 *M* (SD)	Latin-American-born *n =* 815 *M* (SD)	Asian-born *n =* 35 *M* (SD)	Other *n =* 346 *M* (SD)
BA	.222 (.07)	.228 (.09)_a_	.239 (.06)_a_	.238 (.07)	.230 (.08)
SLG	.315 (.11)	.321 (.14)_a_	.347 (.11)_a_	.370 (.20)	.328 (.12)
OPS	.606 (.17)	.612 (.22)_a_	.639 (.17)_a_	.681 (25)	.615 (.20)
OPS+	70.39 (47.25)	69.44 (59.11)	72.78 (45.77)	82.51 (60.04)	70.60 (55.45)
HR^%^	1.40 (1.76)	1.32 (1.71)_a_	1.80 (1.92)_ab_	2.78 (5.65)	1.45 (1.76)_b_
RBI^%^*	10.45 (5.47)	9.72 (6.13)	10.07 (5.74)	11.02 (8.91)	10.05 (6.03)
SO^%^^†^	16.76 (10.79)_ab_	17.37 (13.03)_cd_	20.44 (10.20)_ace_	23.22 (11.42)_bd_	18.07 (13.01)_e_
WAR	4.88 (11.95)	5.35 (13.51)	5.26 (12.69)	4.97 (11.66)	5.13 (12.56)

Note: Values in each row that share the same subscript indicate significant between-group differences using Benjamini-Hochberg adjustment.

^%^ = Percentage of at bats ([Category/Total At Bats] x 100).

* = sample sizes for RBI: Canadian- (*n* = 107), American- (*n* = 8440), Latin-American- (*n* = 815), Asian- (*n* = 35) and Other-born (*n* = 345) players.

^†^ = sample sizes for SO: Canadian- (*n* = 104), American- (*n* = 8409), Latin-American- (*n* = 815), Asian- (*n* = 35) and Other-born (*n* = 343) players.

### Proportion of handedness orientation by birthplace

In the next part of the analysis, we tested the hockey-influence on batting hypothesis by examining whether the proportion of sinister right-handed players born in Canada is higher than those born in the USA, Latin America, Asia and anywhere else. This is a more detailed analysis that builds on previous findings of Cairney et al. [5] which only included players born in Canada, USA, Dominican Republic and South Asia. Cairney et al. [5] also categorized switch hitters as left-handed batters. Findings are displayed in Table 3. The results showed that 41.8% of Canadian-born players are sinister right-handers, which is roughly 10% more than players from Asia, more than twice as many compared to the USA, over five times higher than players from Other regions (i.e., born in countries other than Canada, USA, Latin America and Asia), and eight times as many compared to Latin America (χ^2^ = 170.86, df = 4, *p* < .001; Cramer’s V = .13, *p* < .001).

**Table 3:** Handedness proportions among Canadian-, American-, Latin-, Asian- and Other-born MLB players.

	Birthplace					
Birthplace	Bats right, throws right % (*n*)	Bats right, throws left % (*n*)	Bats left, throws left % (*n*)	Bats left, throws right % (*n*)	Bats left and right, throws right % (*n*)	Bats left and right, throws left % (*n*)
Canada (*n* = 110)	32.7 (36)	0 (0)	18.2 (20)	41.8 (46)	5.4 (6)	1.8 (2)
USA (*n* = 8469)	60 (5078)	0.8 (65)	12.9 (1094)	19.0 (1611)	6.8 (572)	0.5 (49)
Latin America (*n* = 815)	66.5 (542)	0.2 (2)	8.5 (69)	5.1 (42)	19.5 (159)	0.1 (1)
Asia (*n* = 35)	45.7 (16)	0 (0)	17.1 (6)	31.4 (11)	5.7 (2)	0 (0)
Other (*n* = 346)	61.3 (212)	1.2 (4)	12.7 (44)	7.5 (26)	16.5 (57)	0.9 (3)

### Mediation models

In support of the hockey-influence on batting hypothesis, the effect of being Canadian-born on offensive performance is mediated by differences in handedness preference except in the case of rate of HR and RBI production (Table 4). Simply stated, the greater propensity for Canadians to bat left-handed and throw right-handed provides a relative statistical advantage compared to players born in other countries. Closer examination of the indirect effect of handedness preference on the birthplace–offensive performance relationship revealed the higher likelihood for Canadian-born players to be sinister right-handers provides specific statistical advantages when compared against players born in other countries and this effect is largely driven by sinister right-handed Canadian-born players outperforming players from other countries that are dominant right-handers or switch-hitters that throw right-handed (Table 5).

**Table 4:** Mediation analyses of birthplace (Canada vs. World) on offensive performance through handedness orientation (bats left, throws right vs. Other).

Offensive Performance Category	*Z*_a_	*Z*_b_	*Z*_mediation_
BA	6.25	3.00	2.68*
SLG	6.25	2.75	2.49*
OPS	6.25	4.17	3.44*
OPS+	6.25	4.25	3.49*
HR	6.25	1.89	1.79
RBI	6.25	1.19	1.19
SO	6.25	-3.63	-3.11*
WAR	6.25	3.83	3.24*

Note: Positive *Z*_mediation_ values (except for SO) indicate Canadian-born players outperform players from other countries of origin.

* = *Z*_mediation_ is significant at *p <* .05 if it exceeds |1.96| for a two-tailed test with α = .05

**Table 5:** Mediation analyses of birthplace (Canada vs. World) on offensive performance through handedness (Bats left, throws right vs. bats right, throws right; bats right, throws left; bats left, throws left; bats left and right, throws right; bats left and right, throws left).

Offensive Performance Category	Handedness (ref: LR)	*Z*_a_	*Z*_b_	*Z*_mediation_
BA	RR	6.63	4.5	3.70*
	RL	0.00	0.78	0.00
	LL	1.86	-0.33	-0.29
	SR	2.93	1.33	1.16
	SL	-0.44	-0.64	0.22
SLG	RR	6.63	3.75	3.24*
	RL	0.00	0.00	0.00
	LL	1.86	-1.00	-0.80
	SR	2.93	2.2	1.70
	SL	-0.44	-0.06	0.03
OPS	RR	6.63	5.33	4.12*
	RL	0.00	9.13	0.00
	LL	1.86	-0.57	-0.49
	SR	2.93	2	1.59
	SL	-0.44	-0.65	0.23
OPS+	RR	6.63	5.25	4.09*
	RL	0.00	0.71	0.00
	LL	1.86	-0.57	-0.48
	SR	2.93	2.40	1.79
	SL	-0.44	-0.80	0.26
HR	RR	6.63	2.18	2.05*
	RL	0.00	-0.06	0.00
	LL	1.86	-1.50	-1.08
	SR	2.93	3.41	2.17*
	SL	-0.44	-.86	0.27
RBI	RR	6.63	1.12	1.09
	RL	0.00	-0.51	0.00
	LL	1.86	-1.21	-0.92
	SR	2.93	4.05	2.33*
	SL	-0.44	0.28	-0.11
SO	RR	6.63	-3.13	-2.81*
	RL	0.00	0.43	0.00
	LL	1.86	-2.75	-1.48
	SR	2.93	-4.54	-2.42*
	SL	-0.44	-0.85	0.27
WAR	RR	6.63	4.81	3.86*
	RL	0.00	1.60	0.00
	LL	1.86	0.31	0.27
	SR	2.93	0.63	0.59
	SL	-0.44	-0.14	0.06

Note: Positive *Z*_mediation_ values (except for SO) indicate LR outperforms comparison group.

* = *Z*_mediation_ is significant at *p <* .05 if it exceeds |1.96| for a two-tailed test with α = .05

## Discussion

Results from the present study address a number of gaps in our current knowledge regarding factors that influence offensive performance among MLB players. Using the largest archival dataset of MLB statistics available, we found that batting left-handed, regardless of throwing hand dominance, offers a performance advantage over other handedness preference orientations for MLB batters. Owing to the notion that Canadian-born MLB players may be more likely to bat left-handed due to a greater likelihood of being exposed to hockey during development, Cairney et al. [5] proposed the hockey influence on batting hypothesis. Findings further support Cairney’s line of theorizing in that results showed Canadian-born players are more likely to be sinister right-handers compared to players born in other countries. Despite this pattern, being Canadian-born does not provide a direct offensive advantage over players from other countries. Rather, the greater propensity for Canadian-born players to be sinister right-handers has a significant indirect (mediation) effect on offensive performance. Together, results suggest the hockey influence on batting hypothesis extends to relative offensive performance advantages for Canadian-born MLB players.

### Handedness and offensive performance

Recent re-examination of a longstanding question surrounding factors that provide an offensive performance advantage in baseball reversed held beliefs by demonstrating that sinister right-handers, rather than dominant left-handed players, are more likely to be better hitters [4]. Results from the present study provide evidence that offensive performance advantages are not specific to sinister right-handers [4] or dominant left-handers [2], but rather position players who bat left-handed, irrespective of throwing hand dominance. These advantages extend beyond just BA, as other aspects of the game such as hitting for power (i.e., SLG, HR) and getting on base (i.e., OPS, OPS+) were also highest for left-handed MLB players. It should be noted that dominant left-handed players have a trivial, albeit non-significant advantage over sinister right-handers in each offensive category except for SO and WAR. However, given the similarity in offensive performance between sinister right-handers and dominant left-handed batters, previous speculation about biomechanical advantages associated with dominant left-handers [2] and sinister right-handers [4] are not supported. Instead, sinister right-handers and dominant left-handers experience many of the same advantages over right-handed batters (e.g., being closer to first-base, higher likelihood of off-handed match-ups, defensive positioning), which may explain these differences in offensive performance and position left-handed batters for greater success.

### Handedness and birthplace

Despite the apparent offensive advantages for left-handed batters, an overwhelming majority of right-hand dominant MLB players bat right-handed. Although research has shown 80% of right-handed humans place their non-dominant (left) hand at the end of the handle when using bimanual striking tools such as a sledgehammer [11], these handedness preferences do not always hold when transitioning to sport-based tasks as evidenced by only one-third of right-handed hockey players placing their non-dominant hand at the top of the stick [7]. A recent study conducted by Loffing, Solter and Hagemann [12] provided support for these findings by showing a weak correlation between handedness and laterality preference in sport-based bimanual coordination tasks. Cairney et al. [5] have also shown most left-handed batters are right hand dominant (except USA-born players). Our findings generally support this relationship in that over the history of the MLB, left-handed batters from Canada, USA and Asia are more likely to be right-hand dominant, whereas the opposite is the case for Latin-born players and players classified as being born in Other countries. The likelihood of being a sinister right-hander is greatest if a player was born in Canada. Together, our findings suggest factors beyond motor development and learning contribute to laterality preference patterns in baseball.

Drawing from action theory [13], sociocultural and individual factors (i.e., motivation and affect) may contribute to differences in batting laterality preferences specific to hand dominance. For instance, the USA is arguably the largest media market for baseball content worldwide and consumers see that ~70% (excluding switch-hitters) of players bat right-handed. This sociocultural influence coupled with the motivation to adopt a hitting style similar to one’s favourite player may partially explain the overrepresentation of right-hand dominant players that bat right-handed. However, when taking into consideration the popularity of hockey across each State, different trends begin to emerge. Partial support for Cairney and colleagues’ [5] hockey influence on batting hypothesis stems from evidence showing MLB players born in States with higher ice hockey participation are more likely to bat left-handed. Interestingly, data from the 2017–2018 NHL season reveals American-born players are equally likely to shoot left- and right-handed [14], which represents a dramatic difference from the 69% of American-born MLB players that bat right-handed (excluding switch-hitters). Considering conventional hockey coaching practices teach players to use their dominant hand at the top of the stick, this would make a more natural transition to batting with one’s dominant hand at the bottom of the bat as Cairney et al. [5] have illustrated. Therefore, in geographical locations where hockey is less popular, players beginning to play baseball are much more likely to adopt a batting laterality preference that corresponds with their dominant hand.

Conducting a more in-depth investigation of batting laterality preferences among Canadian-born MLB players provides further evidence in support of the hockey influence on batting hypothesis. Current findings demonstrate the greater propensity for Canadian-born MLB players to bat left-handed as reported by Cairney et al. [5] is driven by the fact Canadians are approximately 2.5 times more likely to be sinister right-handers compared to MLB players born in other countries. As previously noted by Cairney et al. [5], hockey provides an interesting context to understand this phenomenon. Data obtained from the 2017–2018 NHL regular season shows over 60% of Canadian-born players shoot left-handed [14]. Given that hockey has been almost synonymous with Canada since the inception of the sport, it is plausible that early hockey exposure is much more common in Canada compared to other countries included in our analysis. In this respect, statistics suggest Canadians are more likely to shoot left-handed, which in turn increases the likelihood of naturally adopting a left laterality batting preference. An opposite cascade of potential events may occur for Americans. That is, initial exposure to baseball increases the propensity to bat right-handed, which may partially account for why 10% more American-born NHL players shoot right-handed. In sum, evidence highlights the potential influential role hockey can play in shaping one’s batting laterality preference and could provide a relative offensive performance advantage in the case of left-handed batters.

### Birthplace, handedness and offensive performance

Based on our mediation analysis, findings suggest the hockey influence on batting hypothesis extends to batting performance. Results showed that being Canadian-born is not directly associated with offensive performance, but rather an indirect relative offensive advantage emerges when adjusting for Canadian-born players’ greater propensity to be sinister right-handers. These effects were largely driven by relative performance advantages over players from other countries that employ batting laterality preferences that correspond with their dominant throwing hand. Taken together, Canadian-born MLB players’ success is partially attributable to laterality preferences that may be established through early exposure to hockey, later positioning these players to capitalize on potential off-handed matchups more often.

While this study addresses many gaps in the literature, several limitations must be acknowledged. First, across the history of the MLB, the proportion of left-handed pitchers has been much smaller than the proportion of left-handed batters. Among years with higher proportions of right-handed pitchers, performance among both left- and right-handed batters has been higher [15]. Therefore, despite findings providing evidence that left-handed batters, regardless of throwing hand dominance, have greater success across a number of advanced batting metrics, the current analysis did not consider whether this effect is confounded by greater exposure to off-handed pitching match-ups. Second, switch-hitting players’ performance was not classified based on the side of the plate they batted from in each plate appearance. Although previous research suggests switch hitters’ splits from both sides of the plate do not differ based on pitching match-ups [16], we were unable to ascertain whether right-hand dominant switch-hitters also experience a relative performance advantage when batting left-handed.

Another limitation relates to the unequal sample sizes across birthplaces and handedness preferences. Specifically, small sample sizes among right-handed (*n* = 71) and switch-hitting batters that throw left-handed (*n* = 55) may have limited the necessary power required to adequately compute these mediation models. With larger sample sizes among these groups, it would be reasonable to expect *Z*-scores reflective of significant indirect effects among these comparisons as well. Fourth, players birthplaces only reflected where they were born and fail to consider the place they grew up. For example, it would be hard to imagine the one player born at sea spent the entirety of his childhood developing in this environment. Thus, future studies should attempt to take into account environmental factors during the development years. Lastly, we still do not know the underlying mechanisms that explain why left-handed batters experience a relative performance advantage. Despite the many logical hypotheses (e.g., more off-handed match-ups, closer to first base), future research is needed to devise experiments to test the mechanisms that explain these effects.

In conclusion, results demonstrate a significant performance advantage among MLB players that bat left-handed, regardless of throwing hand dominance. Despite no direct evidence of a relative performance advantage for Canadian-born MLB players compared to players born elsewhere, the greater propensity for Canadians to be sinister right-handers has an indirect effect on offensive performance that provides Canadians with a relative advantage. In sum, findings further support the hockey influence on batting hypothesis and provide evidence this phenomenon extends to batting performance.
